# *Acinetobacter junii* as an aetiological agent of corneal ulcer

**DOI:** 10.1007/s15010-014-0647-8

**Published:** 2014-07-24

**Authors:** G. Broniek, E. Langwińska-Wośko, J. Szaflik, M. Wróblewska

**Affiliations:** 1 Clinical Ophthalmological Hospital, Medical University of Warsaw, Warsaw, Poland; 2 Chair and Department of Ophthalmology, IInd Faculty of Medicine, Medical University of Warsaw, Warsaw, Poland

**Keywords:** Corneal ulcer, Ulcerative keratitis, *Acinetobacter junii*, Healthcare-associated infections, Community-acquired infections

## Abstract

Rods of the *Acinetobacter* genus are present mainly in the external environment (e.g. water, soil) and in animals, while in humans they may comprise physiological flora. The main pathogenic species is *Acinetobacter baumannii* complex, which constitutes a common cause of nosocomial infections, particularly in patients with underlying diseases and risk factors (e.g. prior broad-spectrum antibiotic therapy, malignancy, central venous catheter, mechanical ventilation); however, infections of the eye caused by strains of *Acinetobacter* spp. are very rare. We report a unique case of community-acquired corneal ulcer caused by *Acinetobacter* non-*baumannii* (possibly *A. junii*), in a patient with no risk factors identified. The case highlights the need for obtaining a sample from the cornea for bacteriological culture in the case of suspected ophthalmic infection as identification of the pathogen, and assessment of its susceptibility profile enables proper antibiotic therapy, improves the outcome and may constitute an eyesight-saving management.

## Introduction

Rods of the *Acinetobacter* genus are Gram-negative coccobacilli. They are present mainly in the external environment (e.g. water, soil) and in animals, while in humans they may comprise physiological flora, particularly in hot and humid climate countries [1]. The main pathogenic species is *Acinetobacter baumannii* complex, which constitutes a common cause of nosocomial infections, particularly in patients with underlying diseases and risk factors (e.g. prior broad-spectrum antibiotic therapy, malignancy, central venous catheter, mechanical ventilation) [1, 2]. However, infections of the eye caused by strains of *Acinetobacter* spp. are uncommon. We present a unique case of community-acquired *Acinetobacter* non-*baumannii* (possibly *A. junii*) corneal ulcer in a patient without recognised risk factors.

## Case report

We report a community-acquired corneal ulcer caused by *A. junii* in a 51-year-old woman with no significant past medical history. She was admitted to the E.R. with a diagnosis of corneal ulcer (ulcerative keratitis) and anterior uveitis of the right eye. For the previous 2 days she complained of flu-like symptoms and pain of the affected eyeball. She was generally in good health, with no history of ophthalmological diseases or treatment. Eight years earlier she was diagnosed with unexplained anaemia, but results of the recent blood tests were within the normal range.

On examination the eyelid and conjunctiva were swollen, with deep and superficial conjunctival injection. There was a large (1/2 of the corneal diameter), white and deep inflammatory infiltrate. A 2-mm hypopyon (accumulation of pus in the anterior chamber) was noticed. Best corrected visual acuity (BCVA) was 0.2.

Corneal scrapings were taken at presentation for microbiological examination. A strain of *A. junii* was isolated in pure culture and identified susceptible to all tested antimicrobials: aminoglycosides (amikacin, gentamicin, tobramycin, neomycin), tetracycline, chloramphenicol, norfloxacin and sulphonamides. The species identification and susceptibility testing was performed in a clinical microbiology laboratory using an automated system VITEK^®^ 2 Compact (bioMerieux). It should be noted, however, that according to the present data the identification of *A. junii* by this automated system is uncertain, in contrast to *A. baumannii* complex [3].

Empiric antibiotic therapy was started on admission and comprised systemic ciprofloxacin (0.2 g b.d.) and topical chloramphenicol ointment (q.i.d.). After obtaining the culture result and an antibiogram of the isolate, this treatment was continued as the strain was susceptible to the antibiotics used in empiric therapy. In the meantime (whilst awaiting the culture results) there was a clinical improvement of the condition of the patient’s eye.

Four days later the corneal ulcer continued to diminish in size and depth, with gradual epithelisation noted in the periphery of the affected area. Descemet’s membrane folds decreased substantially and hypopyon virtually resolved, while fine dust-like deposits were detected on the corneal endothelium. The infection resolved within 2 weeks, with only slight opacity, thinning and single pigment granules still present in the lower nasal quadrant of the cornea. The patient was discharged with BCVA 0.7.

## Discussion

*Acinetobacter* species are opportunistic pathogens causing nosocomial infections [2]. Relatively few infections caused by *Acinetobacter* spp. are community acquired, reported primarily from countries with tropical or subtropical climate, and mainly affected patients with some form of comorbidity or associated with heavy smoking and excess alcohol consumption [4, 5]. Clinical forms of *Acinetobacter* infections include mainly the respiratory tract, bloodstream infections, peritoneum, urinary tract infection, surgical wounds, meningitis, skin and soft tissue infections, while eye infections are rare [2].

The spectrum of ocular infections caused by *Acinetobacter* spp. comprises acute or chronic conjunctivitis, corneal ulcers and dacryocystitis [6, 7]. Exposure of cornea, use of contact lens, penetrating keratoplasty, and immunosuppression have been implicated as predisposing factors to eye infections by *Acinetobacter* [6]. Rods of the genus *Acinetobacter* have been implicated in the aetiology of endophthalmitis and preseptal cellulitis usually preceded by bacteraemia [4, 8–10]. Keratitis caused by *Acinetobacter* spp. is rare and may result from trauma, contact lens wear or ocular surgery, e.g. penetrating keratoplasty—PKP or cataract surgery [4, 6, 8, 11–13]. In a study reporting 750 cases of postoperative ocular infections, *Acinetobacter* spp. comprised 2 % of strains cultured from clinical specimens, being second most common pathogen (after *Pseudomonas*) among Gram-negative bacteria [14]. Interestingly ocular colonisation with *Acinetobacter* may precede its appearance in sputum or blood cultures 1–2 days later [15]. *Acinetobacter* keratitis has also been reported in a patient with chronic lymphocytic leukaemia [16].

Ocular surface colonisation by *Acinetobacter* spp. may be particularly important in sedated intensive care unit patients [17]. Incomplete lid closure leads to drying of the conjunctiva and the corneal epithelium, resulting in lesions ranging from punctate epithelial erosions to a deep ulcer formation [17].

In some cases infections caused by *Acinetobacter* spp. are endogenous, without any obvious predisposing factors [4, 9]. Clinically these ocular infections take a form of endophthalmitis or keratitis with or without ulcer formation [9, 11, 13]. Recently a case of *A. baumannii* endophthalmitis has been reported, which resulted from intravitreal ranibizumab injection [18]. Eye infections may be caused by *A. baumannii* as well as other species such as *A. anitratus* or *A. lwoffii* [4, 8, 11]. Newly described *Acinetobacter* species, e.g. *A. gyllenbergii*-like isolate, have been implicated in ocular infections as well [7].

*Acinetobacter junii* rarely causes infections in humans [19]. In larger analyses from Europe, strains of *A. junii* constitute 3.6–4.8 % of all *Acinetobacter* spp. isolates—12/331 and 9/186, respectively [20, 21]. It mainly affects patients who have had prior antimicrobial therapy, invasive procedures, or malignancy [19]. Out of 34 patients with *A. junii* infections 80 % were hospital-acquired; four patients died (11.4 %) despite adequate antibiotic therapy in three cases [19]. Only one patient had ocular involvement—keratitis. *Acinetobacter junii* has been associated mainly with bacteremia and sepsis in neonates and paediatric oncologic patients [22]. Linde et al. described a case of catheter-related bloodstream infection caused by *A. junii* in an adult oncologic patient [22].

Henao-Martinez et al. [23] described a case of community-onset non-traumatic cellulitis caused by a strain identified as *A. junii*-*johnsonii*. Corneal perforation due to *A. junii* has been reported very rarely [5, 24].

In conclusion it should be noted that *A.* non-*baumannii* species, including *A. junii*, may cause a community-acquired corneal ulceration in an otherwise healthy patient. Ophthalmologists should obtain a sample from the cornea for bacteriological culture in the case of suspected infection as identification of the unique causative pathogen, and its susceptibility profile enables administration of proper antibiotic therapy, improves the outcome and may constitute an eyesight-saving management.
